# Methods to estimate body temperature and energy expenditure dynamics in fed and fasted laboratory mice: effects of sleep deprivation and light exposure

**DOI:** 10.1007/s00360-024-01554-4

**Published:** 2024-04-23

**Authors:** Vincent van der Vinne, Laura E. McKillop, Sian L. Wilcox, James Cantley, Stuart N. Peirson, Steven J. Swoap, Vladyslav V. Vyazovskiy

**Affiliations:** 1 Sleep and Circadian Neurosciences Institute, Department of Physiology and Genetics, University of Oxford, Oxford, UK; 2 Department of Psychology & Neuroscience, Drake University, Des Moines, IA USA; 3 Division of Systems Medicine, School of Medicine, University of Dundee, Dundee, UK; 4 Sleep and Circadian Neurosciences Institute, Nuffield Department of Clinical Neurosciences, University of Oxford, Oxford, UK; 5 Department of Biology, Williams College, Williamstown, MA USA; 6 The Kavli Institute for Nanoscience Discovery, University of Oxford, Oxford, UK

**Keywords:** 3R’s, Body compartments, Intraperitoneal, NC3R, Telemeter, Telemetry

## Abstract

Monitoring body temperature and energy expenditure in freely-moving laboratory mice remains a powerful methodology used widely across a variety of disciplines–including circadian biology, sleep research, metabolic phenotyping, and the study of body temperature regulation. Some of the most pronounced changes in body temperature are observed when small heterothermic species reduce their body temperature during daily torpor. Daily torpor is an energy saving strategy characterized by dramatic reductions in body temperature employed by mice and other species when challenged to meet energetic demands. Typical measurements used to describe daily torpor are the measurement of core body temperature and energy expenditure. These approaches can have drawbacks and developing alternatives for these techniques provides options that can be beneficial both from an animal-welfare and study-complexity perspective. First, this paper presents and assesses a method to estimate core body temperature based on measurements of subcutaneous body temperature, and second, a separate approach to better estimate energy expenditure during daily torpor based on core body temperature. Third, the effects of light exposure during the habitual dark phase and sleep deprivation during the light period on body temperature dynamics were tested preliminary in fed and fasted mice. Together, the here-published approaches and datasets can be used in the future to assess body temperature and metabolism in freely-moving laboratory mice.

## Introduction

Body temperature and metabolic rate in small rodents vary significantly as a function of behavioral state and energetic requirements. In response to energetic challenges such as cold or hunger many small mammals employ daily torpor as an energy saving strategy. Daily torpor in small mammals is characterized by a dramatic drop in metabolic rate resulting in a corresponding decrease in body temperature (Heldmaier et al. 2004). The behavioral appearance of mice during fasting-induced torpor is comparable to that during sleep, with low levels of mobility, a hunched or curled body position, reduced responsiveness to the environment and an overall quiescent state. This behavioral similarity extends also to the electrophysiological (EEG) activity of the brain (Lo Martire et al. 2020; Huang et al. 2021). During the transition into daily torpor, rodents are typically asleep while the EEG amplitude gradually decreases with body temperature (Walker et al. 1979; Harris et al. 1984; DeBoer & Tobler 1996; Huang et al. 2021). During daily torpor in different species of small mammals induced through either short-photoperiod exposure or food-restriction, the EEG of a torpid animal resembles the EEG of a sleeping animal but with a lower amplitude of the slow waves that are typically used to define sleep, and overall lower EEG spectral power, which decreases as a function of brain temperature (DeBoer & Tobler 1996; Huang et al. 2021). However, the relationship between sleep mechanisms and regulation of hypometabolism remain under-investigated, and whether sleep drive affects torpor propensity is unclear (Harding et al. 2018; Kroeger et al. 2018; Yamagata et al. 2021).

Daily torpor is typically described experimentally through body temperature and/or energy turnover measurements. When measuring body temperature, it is important to realize that the temperature varies throughout the body. The golden standard for assessing body temperature in freely moving small mammals is the measurement of core temperature (T_core_) using a chronically-implanted telemeter or logger in the peritoneal cavity. Such an implanted temperature sensor does however require opening of the peritoneum thus resulting in a more invasive surgery compared to a sensor implanted subcutaneously or the completely non-invasive measurement of body temperature by thermal imaging of the skin. Depending on the species/strain/experimental conditions, subcutaneously measured body temperature might be preferrable compared to the measurement of T_core_, though the associated welfare benefits increase the complexity of data interpretation due to the presence of temperature gradients within the body. The body temperature measured subcutaneously (T_sc_) or on the outside of the skin are always lower than T_core_ when ambient temperatures are below T_core_ (Wacker et al. 2012; Meyer et al. 2017; van der Vinne et al. 2020). Our previous assessment of the utility of skin thermography for estimating T_core_ demonstrated that simply adding a constant does not provide an accurate T_core_ estimate (van der Vinne et al. 2020) and this is likely also the case when using T_sc_ to estimate T_core_. Measuring energy expenditure through indirect calorimetry provides a means by which daily torpor can be described in additional detail (Geiser et al. 2014). Indirect calorimetry measurements do however require the animal to be individually housed in a relatively small air-tight box; a set of requirements that complicates these measurements. If it would be possible to estimate energy expenditure with relatively high precision based on T_core_ then this would simplify experimental design substantially.

The experiments described in this manuscript were performed to achieve several objectives. First, by implanting a group of six male mice with both an intraperitoneal and a subcutaneous temperature telemeter, we investigate the relationship between T_core_ and T_sc_ and assess the reliability of a T_core_ estimate based on a linear transformation of T_sc_. Second, we also generated and used a dataset with T_core_ and energy expenditure measurements to describe their relationship (Geiser et al. 2014) and estimate energy expenditure during daily torpor more accurately. Third, by performing nighttime light exposure and daytime sleep deprivation in these same six mice we performed an initial pilot assessment of the interaction between sleep propensity and timing and characteristics of daily torpor. Together, the assessments included in this experiment provides a building block for future studies assessing the relationship between sleep and daily torpor.

## Methods

All animal procedures were approved by the Animal Care and Ethical Review (ACER) Animal Care and Ethical Review Body (AWERB) of the University of Oxford and performed under a UK Home Office license in accordance with all relevant laws and regulations (project license number P828B64BC). Six 3-month-old male wildtype C57Bl/6 J mice were housed individually throughout the study. Two telemeters/loggers (length x diameter: 18 × 9 mm, 1.7 g; Anipill v2, Bodycap, Hérouville Saint-Claire, France) were implanted into each mouse during a single surgery session. A previous check of the factory calibration of a sample of telemeters from the same batch of Anipill telemeters confirmed that calibration of each individual telemeter was not required. The intraperitoneal telemeter implantation was performed as described previously (van der Vinne et al. 2020). The subcutaneously implanted telemeter was inserted through a 1 cm skin incision in the dorsal neck area. A ~ 4 cm subcutaneous pouch was created for this purpose by inserting a blunt forceps and creating a pouch on the left dorsal flank into which the subcutaneous telemeter was inserted before the incision was sutured with 2–3 inverted stitches. Following > 1 week of post-surgical recovery, mice were transferred to open-topped cages. Mice were provided with only a small amount of nesting material so passive infrared locomotor activity sensors were not obscured. Mice were housed in a 12–12 h light–dark cycle at an ambient temperature of 21 ± 1 °C and water was available ad libitum throughout the study. Locomotor activity was recorded using passive infrared sensors and the percentage of time that motion occurred was stored for 10-s intervals (Brown et al. 2020). Energy expenditure was recorded for 24 h in each mouse in each feeding condition. Mice were transferred to a standard individually ventilated cage (IVC) cage (Tecniplast GM550 cage, Tecniplast UK Ltd., London, UK) at least 2 days before indirect calorimetry commenced and this cage was then connected to the tube of the CaloBox gas analyzer (CaloBox, PhenoSys GmbH, Berlin, Germany; ~ 4 s sampling interval; IVC cage volume: 10 L) at the start of the recording ~ 2 h before lights off. Air flow, O_2_%, CO_2_%, and room temperature were continuously measured by the CaloBox and energy expenditure and respiratory quotient were calculated using the CaloBox software using the manufacturer’s calibrations. Calibration of instruments was performed every 15 min using the automated manufacturer’s procedures. Air flow rate during indirect calorimetry measurements was between 1.05 and 1.10 L/min during measurements of ad libitum fed mice and between 1.65 and 1.72 L/min in food restricted mice. Both temperature telemeters were sampled at ~ 30 s intervals. Regular chow food (2016 Teklad global 16% protein diet, Envigo, Blackthorne, UK) was available ad libitum unless indicated otherwise. Daily torpor was induced by restricting mice to a single daily meal of ~ 70% of the daily ad libitum food intake. During food restriction mice were weighed and received their food daily two hours before lights off. Food amounts were calibrated daily to maintain mice at a stable body weight during periods of food restriction and all presented data in food restricted mice were performed under stable conditions with mice maintaining a stable body weight for at least 3 days before the presented recordings. Sleep was manipulated by leaving the regular lights on for one night (resulting in a one-time exposure to 36 h of continuous light) and by sleep depriving the mice during the first six hours of the light phase (Krone et al. 2021). Sleep deprivation was accomplished by providing the mice with novel objects while under observer observation to ensure that novel objects were exchanged whenever a mouse appeared to want to go to sleep.

**Fig. 1:**
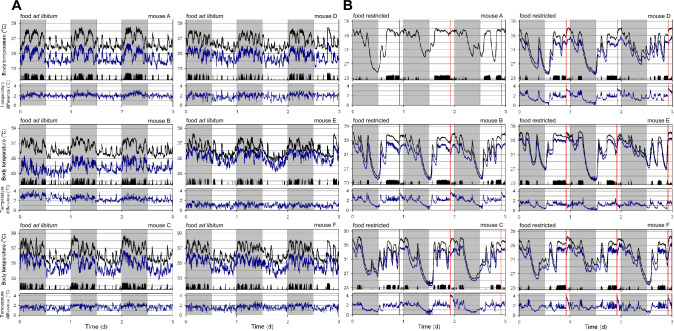
Core and subcutaneous body temperature dynamics in mice fed ad libitum and during food restriction. **A** Body temperature during three days of ad libitum feeding in six male mice. **B** Body temperature during three days of continued food restriction in the same six male mice. For each individual 3-day measurement, core body temperature is plotted by a black line, the subcutaneous temperature is plotted in blue, and black bars at the bottom of each panel depict general locomotor activity. Light and darkness is depicted by the white and grey background, respectively. Temperature differences between the core and subcutaneous temperatures are depicted in the separate window under each main graph. Vertical red lines represent the timing of feeding during food restriction

All assessed variables were re-sampled in 1-min intervals by averaging all measurements occurring during each minute and presented correlations were made using this data with a 1-min interval. Statistical comparisons assessing the effect of time of day were performed on datasets that were re-sampled from 1-min intervals into 30-min intervals by averaging all 1-min values to reduce the complexity in interpreting times-of-day effects between conditions. For the purposes of estimating energy expenditure during daily torpor, T_core_ > 35 °C were excluded from all analyses. Fitting parameters were optimized by minimizing the sum of squares of residuals using the MS Excel Solver function through the GRG Nonlinear solving method. Statistical comparisons were made using a general linear model with daynumber as a random variable for time-of-day comparisons and using an F test in MS Excel to assess other model fits. Visual assessment of residuals confirmed that parametric assessments were appropriate. Missing T_core_ measurements of mouse C during sleep manipulations, due to a drained battery, were replaced by converting T_sc_ measurements using a linear fit that was individually optimized using this mouse’s data presented in Fig. 1B.

## Results

### Estimating T_core_ based on T_sc_

Simultaneous measurements of both T_core_ and T_sc_ for three full days in six male mice generated data to assess the relationship between temperatures in these two body compartments. In the initial assessment (Fig. 1A), mice were provided with ad libitum food availability, and therefore, T_core_ was consistently between 35 and 38 °C. As expected, most locomotor activity occurred during the night, and this was associated with an ~ 1.5 °C elevated T_core_ at this time of day. Furthermore, relatively short bouts of activity during the light phase also resulted in 1–2 °C spikes in T_core_. For each of the six mice throughout the three recording days T_sc_ was consistently lower than T_core_ (Fig. 1A). The average temperature difference between T_core_ and T_sc_ was different in each of the mice (range: 1–2.5 °C). During exposure to food restriction to a single daily meal of ~ 70% of typical food intake the same six mice exhibited regular daily torpor bouts with T_core_ dipping below 25 °C at least once in all of the mice (Fig. 1B). As expected during food restriction (Hut et al. 2012), locomotor activity was mostly shifted to the day with torpor bouts occurring during the late night and early morning. Similar to during ad libitum feeding, T_sc_ was consistently lower than T_core_ (range during activity: 1–3 °C), and a clear pattern emerged with the temperature difference becoming progressively smaller at lower values of T_core_ (minimum difference range: 0.5–1 °C).

**Fig. 2:**
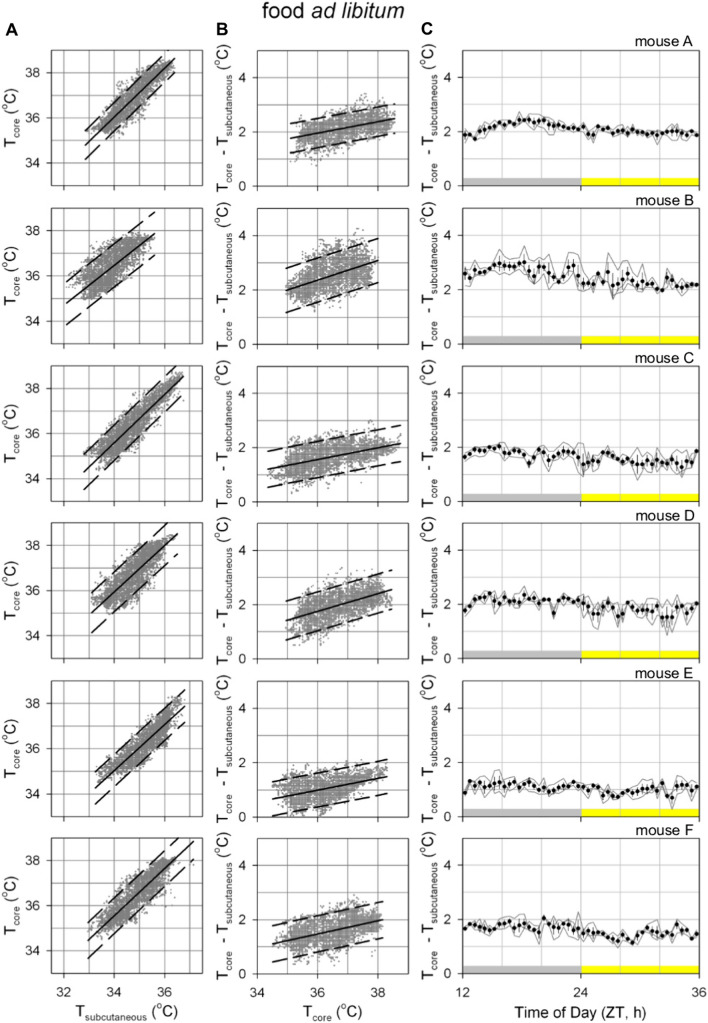
Analyzing the relationship between core and subcutaneous body temperature in ad libitum fed mice. All presented figures are derived from the same data as that presented in Fig. 1. Data from six ad libitum fed mice is depicted in individual graph windows per subdivision with mice A (top) to F (bottom). **A** Core temperature correlates highly with subcutaneous temperature. Solid black lines represent the optimal linear fit of this relationship, dashed lines represent the linear fit line ± 95% confidence interval. **B** The positive slope of the relationship between core temperature and the difference between core and subcutaneous temperature observed in all mice demonstrated that core temperature cannot be properly estimated by simply adding a constant to subcutaneous temperature. **C** Difference between core and subcutaneous temperature at different times of day. Points and error bars represent the mean ± standard error of the mean at each timepoint for the 3-day recording. The grey-yellow bar indicates the light dark cycle. *ZT* zeitgeber time

**Fig. 3:**
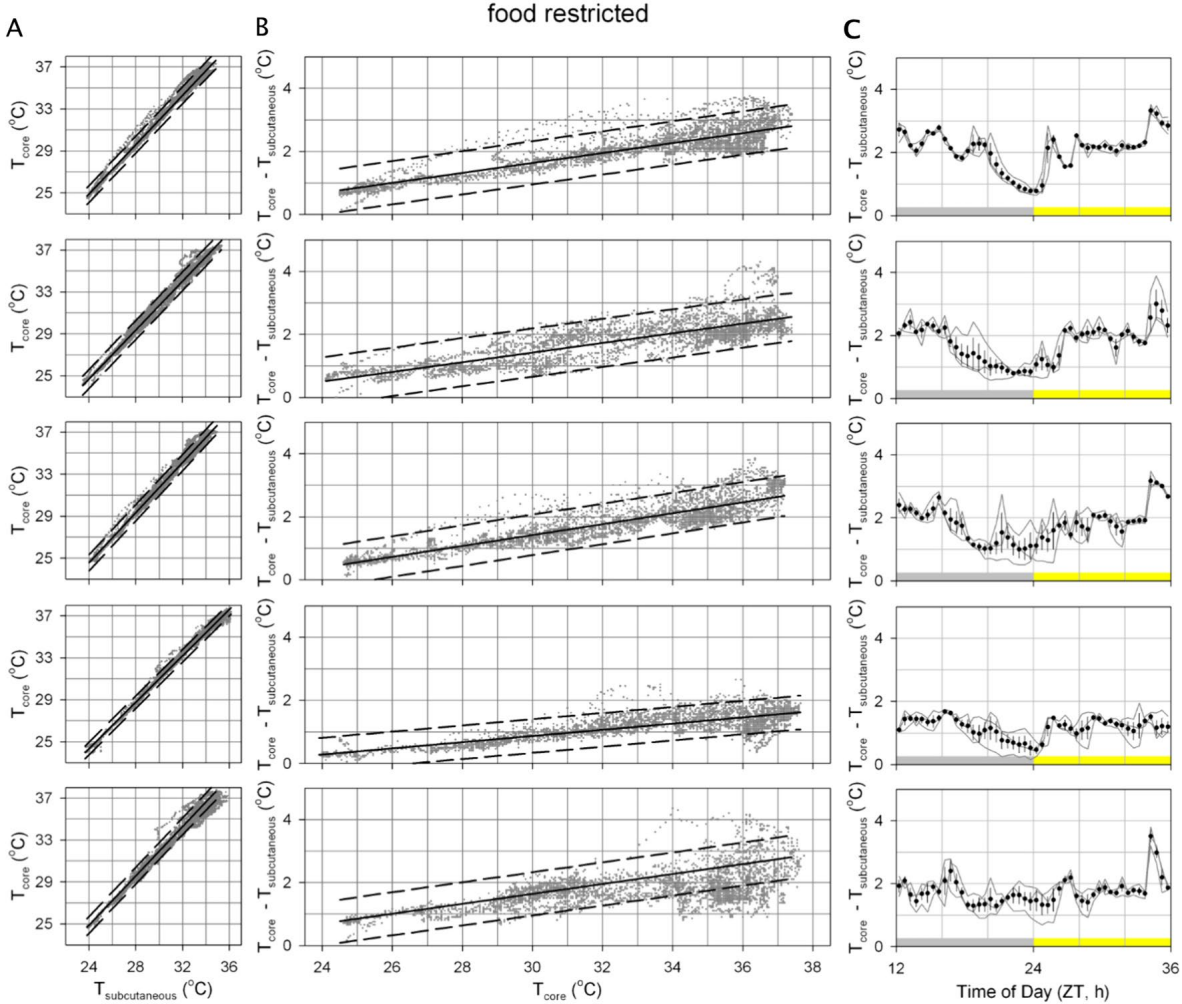
Analyzing the relationship between core and subcutaneous body temperature in food restricted mice. All presented figures are derived from the same data as that presented in Fig. 1. Data from five food restricted mice is depicted in individual graph windows per subdivision with mice B (top) to F (bottom). Drawing conventions as in Fig. 2. **A** Core temperature correlates highly with subcutaneous temperature. **B** The positive slope of the relationship between core temperature and the difference between core and subcutaneous temperature observed in all mice demonstrated that core temperature cannot be properly estimated by simply adding a constant to subcutaneous temperature. **C** Difference between core and subcutaneous temperature at different times of day

The relationship between T_core_ and T_sc_ was further investigated (Figs. 2 and 3). In both ad libitum fed- and food restricted mice, the relationship between T_sc_ and T_core_ appears to be linear (Figs. 2A and 3A) with a slope > 1 (Figs. 2B and 3B; p < 0.05 in each mouse); thus demonstrating that simply adding a constant to T_sc_ does not provide a reliable estimate of T_core_. In line with a slope > 1 for the linear relationship between T_sc_ and T_core_, the difference between these temperatures in *ad* libitum fed mice was also slightly higher during the nighttime active phase (Fig. 2C; all p < 0.05). The occurrence of daily torpor in food restricted mice added additional complexity to this relationship (Fig. 3C), with the temperature difference decreasing dramatically during the late night/early morning when daily torpor typically occurs and an increased temperature difference at the end of the light phase when mice received their daily meal.

**Fig. 4:**
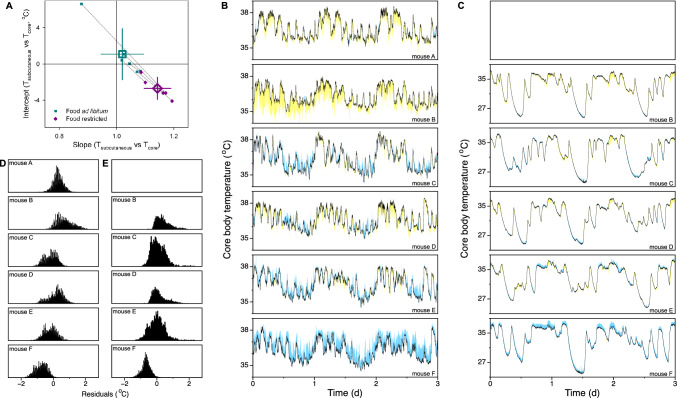
Predicting core body temperature with subcutaneous temperature. **A** The slope and intercept of the optimal linear fit describing the relationship between subcutaneous and core body temperature. Fits were optimized for each individual mouse presented in Fig. 1 (small dots) separately in ad libitum (green) and food restricted (purple) conditions. Large points ± error bars represent the mean ± standard deviation for each feeding condition. **B** **C** Core body temperature measurements (black lines) and the deviation in prediction (yellow/blue) when group-level slope and intercept are used to predict core temperature based on subcutaneous temperature for all six individual mice exposed to ad libitum (**B**) and food restricted conditions (**C**). Yellow and blue colors indicate underestimation and overestimation of core temperature, respectively. **D** **E** Histograms of residuals associated with the core temperature estimations presented in B and C for mice in ad libitum (**D**) and food restricted conditions (**E**)

Given the apparent linear nature of the relationship between T_sc_ and T_core_ in each of the six assessed mice a similar linear relationship appears to be a good way to estimate T_core_ based on T_sc_ measurements. For T_core_ estimates based on T_sc_ measurements to be used as a practical tool in future studies it would be impractical to have to individually calibrate the slope and intercept in each mouse as this would require direct measurement of both T_sc_ and T_core_ in each mouse. Instead, T_core_ is estimated here by multiplying T_sc_ with the mean slope and adding the mean intercept both derived from the six mice measured here (Fig. 4A; ad libitum slope: 1.020, intercept: 1.087 °C; food restriction slope: 1.143, intercept: −2.688 °C). As a preliminary assessment of the utility of this approach to estimate energy expenditure, T_core_ was estimated from T_sc_ measurements for each of the mice using these group-level values (Fig. 4B, C). Since the fit parameters are not optimized for each individual mouse, this results in consistent over- or underestimation of T_core_ in some of the mice. This systematic offset is also seen in the histograms representing the deviations between estimated and measured T_core_ values (Fig. 4D, E) where values do not center on 0 °C, though the mean of all T_core_ estimates fall within a range between −1 and + 1 °C in all of the assessed mice. Analyzing the width of the distribution within each mouse furthermore demonstrates the relatively high consistency of estimates since nearly all estimation errors fall within a range between −1 and + 1 °C of the individual mouse’s mean deviation. Although a further validation assessment of the precision of estimation T_core_ based on T_sc_ measurements and the here derived parameters in a separate group of mouse would be highly advisable, the current analysis indicates that for purposes where the mean T_core_ should be estimated within a range of ± 1 °C and/or within-animal changes within a range of ± 1 °C estimates based on T_sc_ would suffice.

### Estimating energy expenditure based on T_core_

**Fig. 5:**
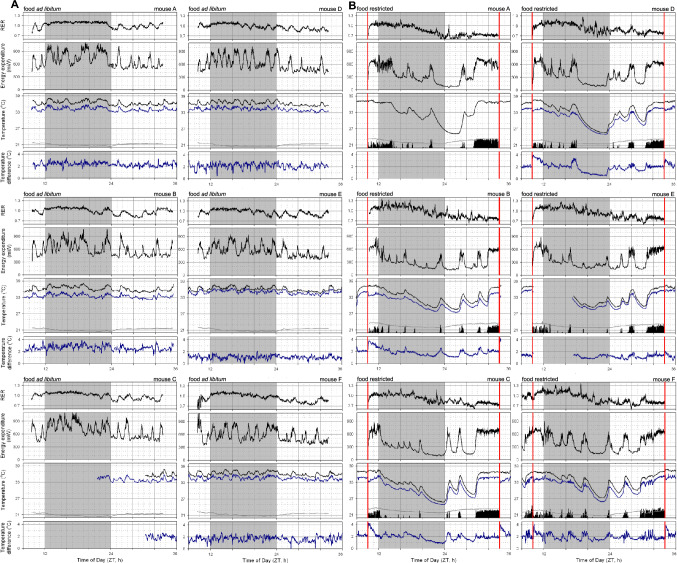
Energy expenditure, respiratory quotient, and body temperature in mice exposed to ad libitum feeding and food restriction. Recordings of six mice recorded for 1 day during ad libitum (**A**) feeding and food restriction (**B**) conditions. Drawing conventions as in Fig. 1. *RER* Respiratory exchange ratio

The estimation of energy expenditure based on T_core_ measurements required the initial recording of energy expenditure and T_core_ over a 1-day period in six mice that were initially fed ad libitum (Fig. 5A) followed by a period of food restriction (Fig. 5B). As expected during ad libitum feeding, T_core_ and energy expenditure were highest during the active phase at night. During food restriction, most locomotor activity as well as peaks in T_core_ and energy expenditure were phase shifted to the light phase. Daily torpor bouts were associated with a rapid decrease in energy expenditure followed by a more gradual reduction in T_core_.

**Fig. 6:**
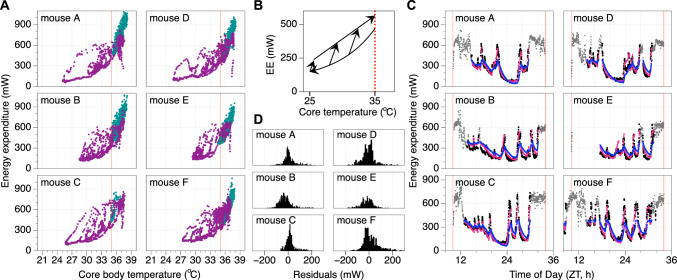
Relationship between core body temperature and energy expenditure in ad libitum fed and food restricted mice. **A** Relationship between core temperature and energy expenditure during ad libitum feeding (green) and food restriction (purple) in six mice. The red vertical line indicates 35 °C core temperature. In ad libitum fed mice core temperature is hardly ever below 35 °C, thus providing a cutoff between ‘normal’ and hypothermic temperatures during food restriction. **B** Schematic depiction of the relationship between core temperature and energy expenditure in food restricted mice during daily torpor. With reducing core temperature, energy expenditure decreases along an exponential trajectory. With increasing core temperature, energy expenditure initially increases rapidly followed by a second stage during which energy expenditure increases slower relative to the increase in core temperature. *EE* energy expenditure. **C** Estimated energy expenditure during daily torpor based on body temperature changes using the relationships described in **B** (red) or using a traditional linear fit (blue) compared to measured energy expenditure (grey). Compared to the traditional model, the novel model is more accurate in describing the substantial changes in energy expenditure observed during daily torpor. T_core_ > 35 °C are excluded from the presented analyses. Thin red vertical lines represent the time of food provision. **D** Histograms of residuals associated with the novel-model fits presented in red in **C**

 Estimation of energy expenditure based on T_core_ requires description of the relationship between T_core_ and energy expenditure (Fig. 6A). A linear fit of this relationship between T_core_ and energy expenditure in ad libitum fed mice resulted in an R^2^ = 0.762 ± 0.037 (mean ± SD). However, a similar linear fit provided a far less accurate description of energy expenditure during daily torpor in food deprived mice (R^2^ = 0.463 ± 0.121; mean ± SD; T_core_ > 35 °C is excluded). Visual inspection of the recordings presented here supports the notion that an alternative ‘hysteresis’ description of the relationship between T_core_ and energy expenditure provides a more reliable estimation of energy expenditure during daily torpor (Geiser et al. 2014; Swoap & Gutilla 2009; Zanetti et al. 2023). In this description the relationship between T_core_ and energy expenditure is partitioned into three distinct components: (1) periods during which T_core_ decreases, (2) an initial stage when T_core_ increases, and (3) a sustained terminal stage of increasing T_core_ during which the increase of energy expenditure is less rapid than during stage 2 (Fig. 6B). The relationship between T_core_ and energy expenditure during stage 1 is described by the exponential function: EE_predicted_ = 4.83 + EXP ((T_core_ + 0.24) / 5.92). The initial increase in energy expenditure (and T_core_) during stage 2 is provided as an iterative increase with an optimized slope: EE_predicted_ (t + 1) = EE_predicted_ (t) + 134.97 * dt [with time described in hours]. The third stage of describing energy expenditure provided by the linear fit of: EE_predicted_ = 565.30 + 34.71 * (T_core_–35 °C). In this model the predicted energy expenditure during daily torpor bouts depends on what stage of the daily torpor bout the mouse is in. Stage 1 is defined by a decreasing body temperature according to a 30-min running average. The subsequent stage 2 has an increasing T_core_ while the predicted energy expenditure is less than that predicted by stage 3, and once the prediction reaches or exceeds the energy expenditure level predicted by the stage 3 formula the prediction will use the stage 3 formula. The energy-estimation described above is implemented in a downloadable MS Excel spreadsheet (https://doi.org/10.6084/m9.figshare.24776868.v1). Using this approach to estimate energy expenditure during daily torpor resulted in an approximately 30% better prediction of the measured energy expenditure (R^2^ = 0.785 ± 0.068; mean ± SD; p < 0.0001) as compared to a simple linear fit. A visual comparison demonstrated that the new way of estimating energy expenditure was much more responsive to the rapid changes in energy expenditure at the initiation and termination of torpor bouts compared to the traditional linear fit (Fig. 6C), resulting in a high reliability of the estimated energy expenditure (Fig. 6D).

### Sleep propensity and daily torpor

**Fig. 7:**
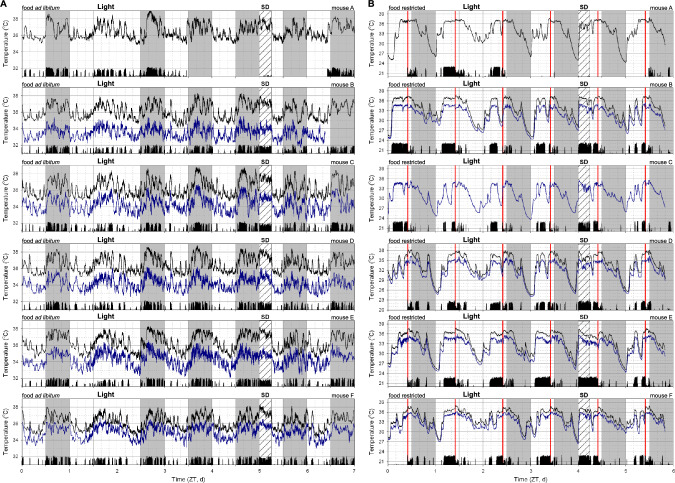
Body temperature during sleep manipulations in mice fed ad libitum and during food restriction. Sleep need was manipulated by leaving light on throughout one night (thus acutely increasing sleep and reducing subsequent sleep pressure) and sleep depriving mice for the first six hours of the light phase (thus acutely preventing sleep and increasing subsequent sleep pressure). Mice were either exposed to ad libitum (**A**) or food restriction conditions (**B**). For each individual 3-day measurement, core body temperature is plotted by a black line, the lower subcutaneous temperature is plotted in blue, and black bars at the bottom of each panel depict general locomotor activity. Light and darkness is depicted by the white and grey background, respectively. Timing of sleep deprivation is indicated by a box labelled SD. Vertical red lines represent the timing of feeding during food restriction

**Fig. 8:**
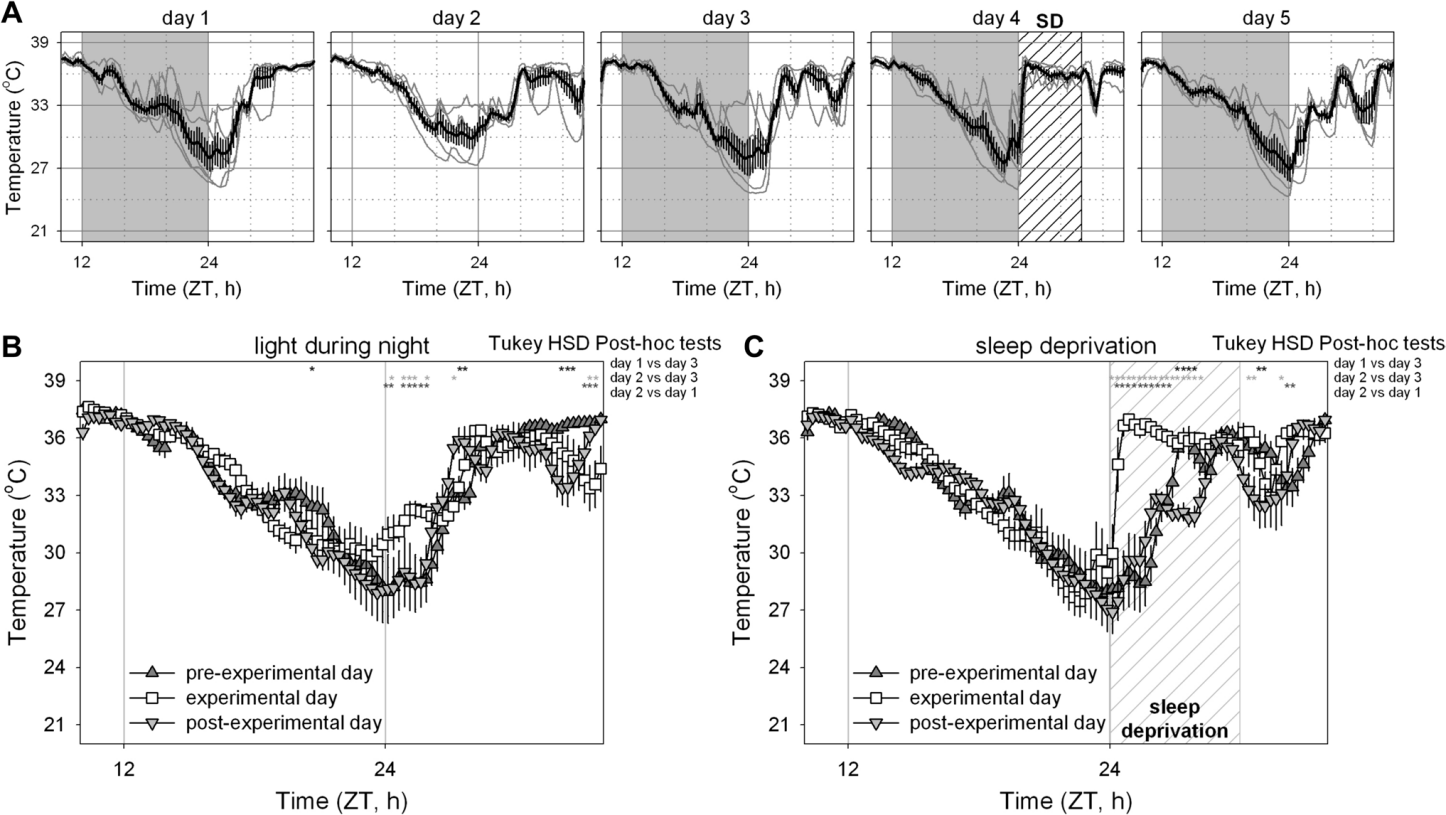
Daily torpor is not substantially altered following manipulations of sleep drive. **A** Individual core body temperature traces as well as mean ± standard error of the mean on the day before, during and after two sleep manipulations in food restricted mice. Lights were kept on throughout the night on day 2 and mice were sleep deprived on day 4. **B** **C** Comparisons of the mean ± standard error of the mean between days for both sleep manipulations. No substantial changes in the timing of daily torpor were observed on the experimental and/or post-experimental day. *ZT* zeitgeber time

The final research question we addressed was whether manipulations known to affect sleep result in changes in body temperature, including the timing and/or depth of daily torpor. Our working hypothesis in these experiments was that elevated sleep pressure would increase the propensity of mice to exhibit daily torpor. To this end, mice were first exposed to a day during which room lights remained on throughout the night; a manipulation designed to acutely increase sleep propensity during the time period when the animals are habitually awake, and consequently reduce sleep pressure during the subsequent day (Fisk et al. 2018). A few days later the same mice were sleep deprived for the first six hours of the light phase; a manipulation designed to increase homeostatic sleep pressure in the subsequent hours. As expected, when performed in ad libitum fed mice, these manipulations resulted only in acute changes of T_core_ (Fig. 7A); a result in line with the interpretation that T_core_ was only altered through changes of the behavioral sleep–wake state (Sela et al. 2021). Subsequent exposure of these same manipulations in food deprived mice did not result in obvious increases in the occurrence, timing, duration and/or depth of daily torpor bouts during or following the manipulations (Fig. 7B). A more in-depth analysis of the body temperature changes on the day before, during, and following the experimental manipulations demonstrated day-specific changes in body temperature (Fig. 8; Day*Time of Day interaction: p < 0.0001 for both manipulations). Specifically, during the days with experimental manipulations, changes in body temperature were observed relative to the preceding day, with continuous light exposure resulting in a less pronounced drop in T_core_ (Fig. 8B). Sleep deprivation, as expected, elevated body temperature in the first hours following lights-on (Fig. 8C). Disruption of daily torpor bouts thus acutely resulted in an increased T_core_. Additional comparisons beyond the immediate effects of sleep manipulations revealed only marginal changes in T_core_. Despite visual observation showing that the mice had increased tendency to fall sleep immediately following six hours sleep deprivation, this increased sleep drive did not result in the induction of a daily torpor bout during the late afternoon (though this time was also when mice were about to be fed their daily meal) nor were the subsequent night’s changes in T_core_ substantially different from those observed on the pre-experimental day. Together these results demonstrate that manipulations known to alter sleep amount or intensity acutely interfered with torpor expression while only minimally altering the torpor expression during the first post-experimental day.

## Discussion

The present paper describes the outcomes of an experiment with multiple distinct objectives, namely, the development of different approaches to quantify the metabolic state of mice under laboratory conditions during daily torpor and to evaluate the effects of sleep manipulations on the expression of daily torpor. The first part of the paper described our approach to optimize the estimation of metabolic markers in mice through the use of other markers that are easier and/or more economical to obtain. Here, our assessment of the estimation of T_core_ based on T_sc_ demonstrated that this provides an estimate that is accurate to within ~ 1 °C when a series of measurements taken from each mouse are compared between animals. When estimating within-individual changes in T_core_, a similar precision of between −1 and + 1 °C is achieved. The ability to estimate T_core_ based on T_sc_ using the equations provided here with this precision makes the presented approach to estimate T_core_ a viable approach for many researchers studying daily torpor in mice under laboratory conditions. This could be especially prescient in light of the potential to reduce the strain placed upon mice with subcutaneous- compared to the intraperitoneal implanted telemeters in terms of health and wellbeing, the elimination of potential physiological changes resulting from intraperitoneal surgery.

The estimation of energy expenditure during daily torpor based on a hysteresis-model using T_core_ measurements (Geiser et al. 2014; Swoap & Gutilla 2009; Zanetti et al. 2023) provides a substantially better estimate compared to traditional models based on a simple linear fit. This improvement is especially pronounced at the initiation and termination of torpor bouts where energy expenditure changes much more rapidly compared to the relatively slow change in T_core_. The great improvement of using the hysteresis approach to estimate energy expenditure, as compared to a linear fit, can visually be appreciated by assessing Mouse A in Fig. 6A. Here, energy expenditure was ~ 150 mW when T_core_ was ~ 30 °C and decreasing, while the same, but increasing, T_core_ values resulted in an energy expenditure of ~ 500 mW. These state-dependent differences in energy expenditure at identical T_core_ values illustrate the importance of not applying a linear correlation to predict energy expenditure, but instead to take the physiological state of the mice into account while estimating energy expenditure during daily torpor. The 30% improvement in the predicted energy expenditure using our ‘hysteresis’ model compared to a traditional linear fit model is substantial, especially when considering that model parameters were not optimized for individual mice and only used intraperitoneal body temperature recordings as a model input. Overall, we consider the here presented method to estimate energy expenditure during daily torpor in mice under laboratory conditions as a means to provide a rough estimate of energy expenditure using the reasonably-accessible method of body temperature telemetry. Potential future studies could increase the accuracy of this prediction through the incorporation of individual-level calibration of model parameters based on indirect calorimetry or double-labelled water measurements. Additionally, future development of this energy expenditure estimation model could incorporate individual activity monitoring. These modifications would however come at the cost of an increased complexity of data collection and analysis.

A limitation of the presented dataset is that the relationship between T_core_ and energy expenditure was only assessed at a constant ambient temperature of 22 °C. This ambient temperature was selected since it is the most-commonly used in laboratory studies of mice. Ideally, future studies will investigate the relationship between T_core_ and energy expenditure at different ambient temperatures and in more ecologically-relevant temperature cycles. Energy expenditure increases at ambient temperatures below 28 and 20 °C in fed and food-deprived mice, respectively (*e.g.* van der Vinne et al. 2014, 2015); thus suggesting that the relationship between T_core_ and energy expenditure will likely change in mice housed below these ambient temperatures. Exposure to daily temperature cycles would also substantially influence energy expenditure during daily torpor bouts, with the greatest energetic benefits of daily torpor to be accrued when torpor bouts coincide with the coldest time of the day (van der Vinne et al. 2015). To conclude, it is worth highlighting that the state-dependent hysteresis relationship describing energy expenditure as a function of T_core_ during daily torpor appears similar in a range of different species undergoing daily torpor (Geiser et al. 2014); thus lending credence to the validity of the approach quantified above.

The final objective of the presented experiment was to evaluate whether manipulations known to alter sleep propensity affect the dynamics of body temperature and specifically daily torpor expression. Sleep and daily torpor share important behavioral similarities, but their relationship remains to be fully characterized. Typically, bouts of daily torpor are preceded by intense non-REM sleep (Huang et al. 2021), and sleep intensity further increases following sleep deprivation, as if the animals were sleep deprived (DeBoer & Tobler 1996). As has previously been well described, EEG power density decreases as a function of body temperature (Huang et al. 2021), which complicates comparison of daily torpor and sleep states directly. Despite these complexities, overlapping neurophysiological mechanisms underlying sleep and daily torpor raise the question whether manipulating sleep alters daily torpor propensity. To this end, we employed acute light exposure during the habitual dark period and sleep deprivation during the first six hours of the light phase to manipulate sleep propensity in our mice.

Although only a limited set of conclusions can be drawn from this pilot study, our data does demonstrate that a (presumed) physiological increase in sleep pressure through six hours of sleep deprivation did not result in an induction of daily torpor in the remaining six hours of the light phase. The absence of an immediate induction of daily torpor following the end of sleep deprivation (when sleep pressure is highest) might very well have been the result of the daily food provisioning just a few hours later, resulting in the effects of food anticipation occurring at the same time that sleep pressure was highest. Future experiments should therefore vary the timing of sleep deprivation relative to the timing of food access to further address whether homeostatic sleep pressure affects torpor propensity or characteristics. One potential caveat in any such experiment would be that since energy expenditure is low during sleep, sleep deprivation will automatically present a metabolic challenge thus complicating the interpretation of any such study. A further limitation of the current study was the lack of objectively-recorded sleep, using conventional EEG and EMG methodology, which would ideally be used to answer the present research question. Finally, exposure to light throughout the night, presumably resulting in increased sleep during this manipulation, did not result in a systematic change in torpor timing or characteristics, though high-interindividual variability was apparent; thus potentially obscuring a disruptive effect of light during the typical dark phase on daily torpor expression. Overall, the data presented here suggest that high sleep pressure during the light phase or increased sleep propensity during the night are not sufficient to acutely induce daily torpor bouts in food-restricted laboratory mice. Future studies will hopefully continue to elucidate the mechanistic role (in conjunction with other factors) of differences in sleep pressure in modulating the timing of daily torpor.

## Data Availability

Data presented in this paper is available for download An MS Excel file with the formulas to estimate energy expenditure based on T_core_ can be downloaded at (10.6084/m9.figshare.24776868.v1); data presented in Figures 1 and 4 can be downloaded in condensed (10.6084/m9.figshare.24776898.v1) or extended form (10.6084/m9.figshare.24776928.v1).
